# Inflammation and renal function decline in chronic coronary syndrome: a prospective multicenter cohort study

**DOI:** 10.1186/s12872-023-03565-5

**Published:** 2023-11-16

**Authors:** Ting-Wei Kao, Chin-Chou Huang, Hsin-Bang Leu, Wei-Hsian Yin, Wei-Kung Tseng, Yen-Wen Wu, Tsung-Hsien Lin, Hung-I Yeh, Kuan-Cheng Chang, Ji-Hung Wang, Chau-Chung Wu, Jaw-Wen Chen

**Affiliations:** 1 Department of Medical Education, Taipei Veterans General Hospital, Taipei, Taiwan; 2 School of Medicine, National Yang Ming Chiao Tung University, Taipei, Taiwan; 3 Department of Internal Medicine, National Taiwan University Hospital, Taipei, Taiwan; 4 Division of Cardiology, Department of Medicine, Taipei Veterans General Hospital, Taipei, Taiwan; 5 Institute of Pharmacology, College of Medicine, National Yang Ming Chiao Tung University, Taipei, Taiwan; 6 Healthcare and Services Center, Taipei Veterans General Hospital, Taipei, Taiwan; 7 Division of Cardiology, Heart Center, Cheng-Hsin General Hospital, Taipei, Taiwan; 8 Department of Medical Imaging and Radiological Sciences, I-Shou University, Kaohsiung, Taiwan; 9 Division of Cardiology, Department of Internal Medicine, E-Da Hospital, Kaohsiung, Taiwan; 10 Cardiology Division of Cardiovascular Medical Center, Far Eastern Memorial Hospital, New Taipei City, Taiwan; 11 Division of Cardiology, Department of Internal Medicine, Kaohsiung Medical University Hospital and Kaohsiung Medical University, Kaohsiung, Taiwan; 12 Mackay Memorial Hospital, Mackay Medical College, New Taipei City, Taiwan; 13 Division of Cardiology, Department of Internal Medicine, China Medical University Hospital, Taichung, Taiwan; 14 Graduate Institute of Clinical Medical Science, China Medical University, Taichung, Taiwan; 15 Department of Cardiology, Buddhist Tzu-Chi General Hospital, Tzu-Chi University, Hualien, Taiwan; 16 Division of Cardiology, Department of Internal Medicine, National Taiwan University Hospital and National Taiwan University College of Medicine, Taipei, Taiwan; 17 Graduate Institute of Medical Education & Bioethics, College of Medicine, National Taiwan University, Taipei, Taiwan; 18 Department of Medical Research and Division of Cardiology, Department of Internal Medicine, Taipei Medical University Hospital, Taipei, Taiwan

**Keywords:** Biomarker, Chronic coronary syndrome, Coronary artery Disease, Inflammation, Renal function

## Abstract

**Background:**

Renal function decline is a frequently encountered complication in patients with chronic coronary syndrome. Aside from traditional cardiovascular risk factors, the inflammatory burden emerged as the novel phenotype that compromised renal prognosis in such population.

**Methods:**

A cohort with chronic coronary syndrome was enrolled to investigate the association between inflammatory status and renal dysfunction. Levels of inflammatory markers, including high-sensitivity C-reactive protein (hs-CRP), tumour necrosis factor-α (TNF-α), adiponectin, matrix metalloproteinase-9, interleukin-6, lipoprotein-associated phospholipase A2, were assessed. Renal event was defined as > 25% decline in estimated glomerular filtration rate (eGFR). Inflammatory scores were calculated based on the aggregate of hs-CRP, TNF-α, and adiponectin levels.

**Results:**

Among the 850 enrolled subjects, 145 patients sustained a renal event during an averaged 3.5 years follow-up. Multivariate analysis with Cox regression suggested elevations in hs-CRP, TNF-α, and adiponectin levels were independent risk factors for the occurrence of a renal event. Whereas, Kaplan-Meier curve illustrated significant correlation between high TNF-α (*P* = 0.005), adiponectin (*P* < 0.001), but not hs-CRP (*P* = 0.092), and eGFR decline. The aggregative effect of these biomarkers was also distinctly correlated with renal events (score 2: *P* = 0.042; score 3: *P* < 0.001).

**Conclusions:**

Inflammatory burden was associated with eGFR decline in patients with chronic coronary syndrome.

**Supplementary Information:**

The online version contains supplementary material available at 10.1186/s12872-023-03565-5.

## Background

Renal dysfunction is a pivotal yet less investigated complication in patients with coronary artery disease (CAD). Despite advancements in revascularization and antithrombotic regimens, integrated care for patients with coronary illness remained suboptimal, particularly due to inadequate monitoring and management of the comorbidities. Due to shared risk factors and common underlying etiologies, decline in creatinine clearance subsequent to CAD accounts for a major clinical burden. The rate of acute kidney injury in patients with CAD was 2.6% in the National Cardiovascular Data Registry, and a considerable proportion advanced to chronic kidney disease (CKD) dependent on renal replacement therapy [1]. Furthermore, CAD predominantly necessitated percutaneous coronary intervention (PCI) for revascularization, and contrast exposure during the procedure further predisposed renal dysfunction. These marked the clinical unmet need to delineate the cardiorenal interplay and improve the overall outcome in this population.

Inflammatory burden emerged as a critical factor which compromises renal prognosis after acute myocardial infarction (AMI). Previous study has suggested a high inflammatory status is correlated with plaque instability and thromboembolic events [2]. In addition, Stuveling et al. first proposed that elevated C-reactive protein (CRP) levels were independently associated with decreased renal filtration rate in subjects without diabetes mellitus (DM) [3]. The relationship was further suggested secondary to the abundance of body fat mass. In addition, CRP was pinpointed to exacerbate renal dysfunction among smokers, who are at peculiar risk of developing CAD [4]. Since cardiac morbidity and renal dysfunction are correlated bidirectionally, high systematic inflammation was hypothesized involving in the crosstalk. Another debate concerns whether cumulative exposure to CRP predisposes to aggravation of renal dysfunction. In a large cohort study, the legacy effect of CRP in a four-year follow-up study showed a significant correlation with renal function decline [5]. Extended spectrum of other inflammatory markers has also been under investigation to indicate post-infarction inflammatory status. Together, these findings highlighted the central role of inflammation affecting cardiorenal physiology after coronary event.

How inflammatory burden alters renal dysfunction with CAD after intervention remains elusive. Although major adverse cardiac events (MACEs) after CAD have been extensively delineated, the paucity of documentations upon renal outcomes warrant detailed investigations. Therefore, the study aimed to elucidate the decline in renal function secondary to active inflammatory status. An inflammatory score aggregated by the level of each marker was developed to predict renal event rate and to investigate the correlation between inflammatory status and renal function decline in patients with chronic coronary syndrome.

## Methods

### Study design

**Fig. 1:**
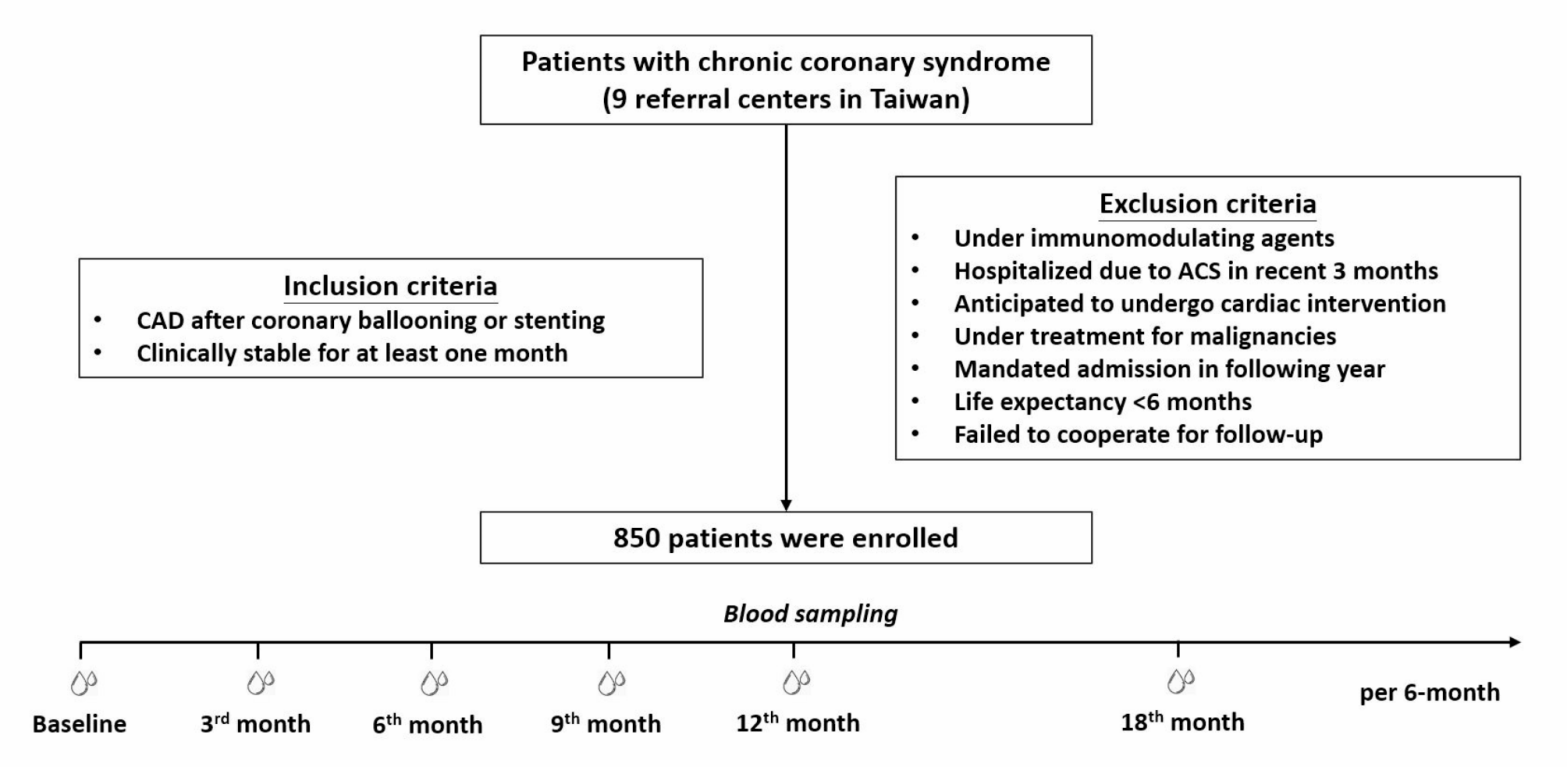
Flowchart of the study design. A cohort of patients with chronic coronary syndrome was included from nine medical centers in Taiwan. The panels of biochemistry, inflammatory markers, and renal function were obtained at baseline and during follow-up. ACS, acute coronary syndrome; CAD, coronary artery disease

The research is based on the ‘*Development of New Biosignatures for Atherosclerosis Cardiovascular Diseases*’ study, a multicenter cohort registry that prospectively enrolled a series of patients with CAD after PCIs. The study protocol has been published previously [6]. The patients with chronic coronary syndrome were included in a nation-based manner from nine tertiary referral centers in Taiwan during 2012 to 2017. The study flowchart is shown in Fig. 1.

### Patient

Subjects who fulfilled the inclusion criteria were enrolled: presence of a significant CAD history after at least one PCI with coronary ballooning or stenting and remained clinically stable under medical treatment for at least one month before this enrollment. Individuals who met any of the following circumstances were excluded: under treatment with non-steroid anti-inflammatory drug, steroid, disease modifying antirheumatic drug, or other biological immunosuppressants at enrollment or any time-point during follow-up, with underlying autoimmune diseases, had been hospitalized due to acute coronary syndrome in the recent three months, anticipated to receive coronary or other cardiac interventions in the following one year, undergoing therapy for compelling malignancy, mandatorily hospitalized for other systemic diseases in the following one year, with a life expectancy of less than six months, and failed to cooperate with clinical follow-up.

The study complied with the principles of the Declaration of Helsinki. Approvals from the independent ethics committees and review boards of each hospital were obtained (IRB: AS-IRB01-19007). Informed consent was obtained from all subjects before participating in this study.

### Clinical assessment

Demographic information was documented in accordance to standardized protocol by a specially trained nurse from the chart or structured questionnaires. The blood pressure (BP) values after being well-rested were measured using an electronic BP monitor operated by a trained nurse and recorded as the average of three consecutive measurements at the outpatient clinic *ante meridiem*. Hypertension was defined as a BP level exceeding 140/90 mmHg or taking antihypertensive agents. All subjects were followed on an outpatient basis at the respective institutions.

### Biomarker measurement

The levels of inflammatory biomarkers with high-sensitivity CRP (hs-CRP), adiponectin, matrix metalloproteinase-9 (MMP-9), interleukin-6 (IL-6), lipoprotein-associated phospholipase A2 (Lp-PLA2), and tumor necrosis factor-α (TNF-α) as well as baseline serum chemistry, including N-terminal pro-brain natriuretic peptide (NT-pro BNP), and uric acid levels, were assessed at enrollment. Whereas, renal function with creatinine was evaluated at the initial visit, every three months in the first year, and at six-month intervals subsequently. Peripheral blood samples (20 ml) were collected for biochemical assessments. The samples were centrifuged before the sera were thawed for assessment.

### Renal event

Estimated glomerular filtration rate (eGFR) was derived from serum creatinine level and demographic parameters based on Modification of Diet in Renal Disease (MDRD) [7] and Chronic Kidney Disease Epidemiology Collaboration (CKD-EPI) Eq [8]. A renal event was defined as a decline of over 25% from the baseline eGFR according to previous guideline [9].

### Inflammatory score

A scoring system was established based on the aggregate of each biomarker level to reflect the burden of inflammation. The respective levels of inflammatory biomarkers (hs-CRP, adiponectin, and TNF-α) which exceeds 50th percentile were assigned one point based on previous literature [10, 11]. Inflammatory score was yielded from the summation thereof.

### Statistical analysis

Statistical Package for Social Sciences software (Version 21.0, SPSS Inc., Chicago, IL, USA) was used for analysis. Continuous variables were presented as mean ± standard deviation, while categorical parameters were presented as numbers with percentages. The Kaplan–Meier curve and log-rank test were employed to assess the renal event rate based on individual and aggregated inflammatory markers. Multivariate analysis in conjunction with Cox proportional hazard regression model was used to evaluate the independent association between inflammatory markers and renal function decline. Adjustment was performed for potential confounding factors, including age, sex, body mass index (BMI), BP, hypertension, DM, use of antihypertensive agents, baseline eGFR, and expression of inflammatory markers. Hazard ratios (HRs) and 95% confidence intervals (CIs) of each parameter were presented. A two-sided *P* value less than 0.05 was considered statistically significant.

## Results

A total of 850 patients with chronic coronary syndrome were enrolled (Fig. 1). The mean age of the subjects was 66.3 ± 12.4 years, and 85.8% subjects were males. The average BMI was 26.2 ± 3.6 kg/m^2^. The initial BP were at 128.7 ± 16.4/73.9 ± 12.2 mmHg in average, and 65.9% subjects were considered hypertensive. DM was rendered to 36% population, while in the predominant absence of hyperuricemia. Among antihypertensive agents, angiotensin-converting enzyme inhibitors/angiotensin receptor blockers, beta-blockers, calcium channel blockers, and diuretics were used in 65.3%, 59.8%, 40.4%, and 17.9% individuals, respectively. As for baseline renal function, the creatinine level was at 1.1 ± 0.3 mg/dL and eGFR at 77.6 ± 28.8 mL/min/1.73 m^2^. Plasma levels of the inflammatory markers, including hs-CRP, adiponectin, and TNF-α levels, were summarized (Table 1).

**Table 1:** Baseline characteristics of enrolled subjects

	All (n = 850)	eGFR* decline (n = 145)	eGFR* maintained (n = 705)	*P-value*
Age, years	66.3 ± 12.4	69.7 ± 13.0	65.5 ± 12.1	< 0.001
Male, n(%)	729 (85.8%)	119 (82.1%)	610 (86.5%)	0.162
BMI, kg/m^2^	26.2 ± 3.6	25.9 ± 3.6	26.3 ± 3.6	0.244
SBP, mmHg	128.7 ± 16.4	130.6 ± 18.8	128.4 ± 15.8	0.141
DBP, mmHg	73.9 ± 12.2	71.6 ± 14.6	74.4 ± 11.6	0.014
HTN, n(%)	560 (65.9%)	100 (69.0%)	460 (65.2%)	0.390
DM, n(%)	306 (36.0%)	65 (44.8%)	241 (34.2%)	0.015
Smoking, n(%)	477 (56.1%)	75 (51.7%)	402 (57.0%)	0.242
ACEI/ARB, n(%)	555 (65.3%)	104 (71.7%)	451 (64.0%)	0.074
B-blocker, n(%)	508 (59.8%)	95 (65.5%)	413 (58.6%)	0.121
CCB, n(%)	343 (40.4%)	71 (49.0%)	272 (38.6%)	0.020
Diuretics, n(%)	152 (17.9%)	34 (23.4%)	118 (16.7%)	0.055
Statins, n(%)	631 (74.2%)	104 (71.7%)	527 (74.8%)	0.448
LVEF < 40%, n(%)	49 (5.8%)	10 (6.9%)	39 (5.5%)	0.521
Creatinine, mg/dL	1.1 ± 0.3	1.1 ± 0.4	1.1 ± 0.3	0.065
eGFR ^*^, mL/min /1.73m^2^	77.6 ± 28.8	79.7 ± 48.8	77.2 ± 22.5	0.356
Uric acid, mg/dL	6.5 ± 1.6	6.7 ± 1.8	6.4 ± 1.6	0.069
hs-CRP, mg/dL	0.3 ± 0.9	0.5 ± 1.6	0.3 ± 0.7	0.030
TNF-α, pg/mL	4.0 ± 4.7	4.8 ± 6.5	3.8 ± 4.2	0.014
Adiponectin, ng/mL	8096.8 ± 13341.3	11479.4 ± 19574.1	7401.0 ± 11551.6	0.001
NT-pro BNP, pg/mL	418.0 ± 955.3	706.9 ± 1856.1	358.6 ± 612.5	< 0.001
Follow up duration, years	3.5 ± 1.9	3.6 ± 1.9	3.5 ± 1.9	0.618

* eGFR is calculated by Modification of Diet in Renal Disease (MDRD) equation. ACEI, angiotensin converting enzyme inhibitor; ARB, angiotensin receptor blocker; BMI, body mass index; CCB, calcium channel blocker; DBP, diastolic blood pressure; DM, diabetes mellitus; eGFR, estimated glomerular filtration rate; hs-CRP, high-sensitivity C-reactive protein; HTN, hypertension; LVEF, left ventricular ejection fraction; NT-pro BNP, N-terminal pro-brain natriuretic peptide; SBP, systolic blood pressure; TNF-α, tumor necrosis factor-α

During the mean follow-up period of 3.5 ± 1.9 years, 145 patients experienced a renal event. In conjunction with traditional cardiovascular risk factors, including age (*P* < 0.001), diastolic BP (*P* = 0.014), and DM (*P* = 0.015), the level of NT-pro BNP showed significantly difference between patients with or without subsequent renal events (*P* < 0.001). Statin use and the proportion of reduced left ventricular ejection fraction (< 40%) were similar in the two groups. Increased levels of the following inflammatory markers were associated with over 25% eGFR decline: serum hs-CRP (*P* = 0.030), TNF-α (*P* = 0.014), and adiponectin (*P* = 0.001). In contrast, there was no association between IL-6, MMP-9, Lp-PLA2 levels and renal function decline (Table 1). Angiographic characteristics were available for a total of 687 subjects, including 110 patients with declined eGFR and 577 patients with maintained eGFR. No statistically significant differences were observed in the angiographic characteristics between the two groups (Supplementary Table 1).

**Fig. 2:**
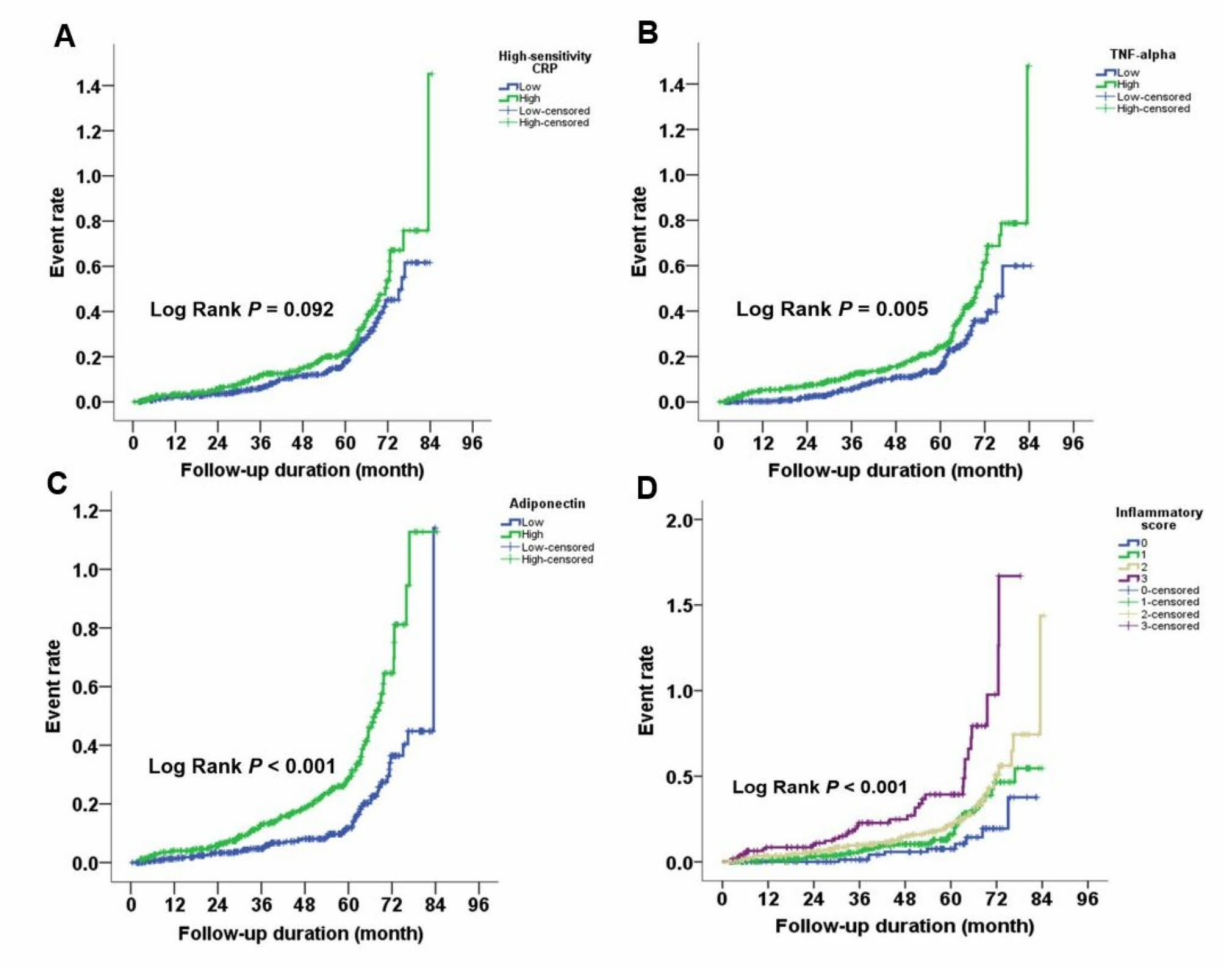
Correlation between the level of inflammatory markers and renal events. The Kaplan–Meier curves of (**A**) tumor necrosis factor-α, (**B**) adiponectin, (**C**) high-sensitivity C-reactive protein, and (**D**) inflammatory score illustrated high inflammatory status was intertwined with deteriorating renal function

In Kaplan–Meier survival curve and log-rank test, increased levels of TNF-α (*P* = 0.005) and adiponectin (*P* < 0.001), but not of hs-CRP (*P* = 0.092), were correlated with renal function decline (Fig. 2A C). After the adjustment of potential confounding factors, the multivariate analysis with Cox regression delineated hs-CRP (HR = 1.194, 95% CI = 1.083–1.316, *P* < 0.001), TNF-α (HR = 1.027, 95% CI = 1.001–1.053, *P* = 0.040), and adiponectin (HR = 1.008, 95% CI = 1.000–1.015, *P* = 0.041) with compelling associations regarding renal function decline (Table 2). Other involved clinical parameters included age, DM status, and baseline eGFR. Interestingly, the level of NT-pro BNP was distinctly elevated in patients with compromised renal prognosis (HR = 1.101, 95% CI = 1.028–1.180, *P* = 0.006) (Table 2).

**Table 2:** Association between levels of inflammatory markers and renal events by multivariate analysis with Cox regression

	HR (95% CI)	*P-value*		HR (95% CI)	*P-value*		HR (95% CI)	*P-value*		HR (95% CI)	*P-value*
Age, years	1.034 (1.016–1.052)	< 0.001	Age, years	1.030 (1.013–1.048)	0.001	Age, years	1.031 (1.013–1.048)	0.001	Age, years	1.031 (1.014–1.049)	< 0.001
Sex (male vs. female)	0.711 (0.454–1.112)	0.135	Sex (male vs. female)	0.743 (0.475–1.161)	0.192	Sex (male vs. female)	0.744 (0.477–1.160)	0.192	Sex (male vs. female)	0.716 (0.458–1.122)	0.145
BMI, kg/m^2^	0.990 (0.943–1.041)	0.704	BMI, kg/m^2^	0.989 (0.942–1.039)	0.665	BMI, kg/m^2^	0.989 (0.941–1.039)	0.658	BMI, kg/m^2^	0.991 (0.944–1.041)	0.720
DBP, mmHg	0.991 (0.976–1.007)	0.265	DBP, mmHg	0.993 (0.978–1.008)	0.364	DBP, mmHg	0.993 (0.977–1.008)	0.344	DBP, mmHg	0.995 (0.979–1.010)	0.491
HTN (yes vs. no)	0.841 (0.558–1.267)	0.407	HTN (yes vs. no)	0.914 (0.603–1.386)	0.672	HTN (yes vs. no)	0.869 (0.576–1.309)	0.501	HTN (yes vs. no)	0.830 (0.551–1.251)	0.374
DM (yes vs. no)	1.880 (1.323–2.670)	< 0.001	DM (yes vs. no)	1.836 (1.296–2.601)	0.001	DM (yes vs. no)	1.814 (1.281–2.569)	0.001	DM (yes vs. no)	1.833 (1.292–2.599)	0.001
ACEI/ARB (yes vs. no)	0.929 (0.626–1.379)	0.715	ACEI/ARB (yes vs. no)	0.904 (0.610–1.340)	0.616	ACEI/ARB (yes vs. no)	0.899 (0.606–1.335)	0.597	ACEI/ARB (yes vs. no)	0.948 (0.636–1.412)	0.792
B-blocker (yes vs. no)	1.330 (0.926–1.909)	0.122	B-blocker (yes vs. no)	1.327 (0.926–1.904)	0.124	B-blocker (yes vs. no)	1.388 (0.968–1.992)	0.075	B-blocker (yes vs. no)	1.380 (0.959–1.986)	0.083
CCB (yes vs. no)	1.246 (0.863–1.800)	0.240	CCB (yes vs. no)	1.278 (0.885–1.846)	0.190	CCB (yes vs. no)	1.274 (0.883–1.838)	0.195	CCB (yes vs. no)	1.347 (0.931–1.948)	0.114
Diuretics (yes vs. no)	0.827 (0.531–1.287)	0.400	Diuretics (yes vs. no)	0.900 (0.584–1.385)	0.631	Diuretics (yes vs. no)	0.899 (0.585–1.381)	0.627	Diuretics (yes vs. no)	0.895 (0.581–1.378)	0.615
eGFR*, mL/min /1.73m^2^	1.006 (1.002–1.010)	0.004	eGFR*, mL/min /1.73m^2^	1.006 (1.002–1.010)	0.005	eGFR*, mL/min /1.73m^2^	1.005 (1.001–1.009)	0.027	eGFR*, mL/min /1.73m^2^	1.006 (1.002–1.010)	0.004
hs-CRP	1.194 (1.083–1.316)	< 0.001	TNF-α	1.027 (1.001–1.053)	0.040	Adiponectin x 10^3^	1.008 (1.000–1.015)	0.041	NT-pro BNP x 10^3^	1.101 (1.028–1.180)	0.006

* eGFR is calculated by Modification of Diet in Renal Disease (MDRD) equation. ACEI, angiotensin converting enzyme inhibitor; ARB, angiotensin receptor blocker; BMI, body mass index; CCB, calcium channel blocker; CI, confidence interval; DBP, diastolic blood pressure; DM, diabetes mellitus; eGFR, estimated glomerular filtration rate; HTN, hypertension; HR, hazard ratio; hs-CRP, high-sensitivity C-reactive protein; NT-pro BNP, N-terminal pro-brain natriuretic peptide; TNF-α, tumor necrosis factor-α

Inflammatory score was calculated according to the baseline levels of these three markers demonstrated with pronounced association, i.e., hs-CRP, TNF-α, and adiponectin, The aggregated inflammatory burden was significant in predicting renal events (*P* < 0.001) (Fig. 2D). Multivariate analysis with Cox regression model also revealed that subjects with higher inflammatory score exhibited a greater risk of eGFR decline (score 2: HR = 2.120, 95% CI = 1.029–4.366, *P* = 0.042; score 3: HR = 4.649, 95% CI = 2.188–9.874, *P* < 0.001) (Table 3).

**Table 3:** Inflammatory scores and renal function decline by multivariate analysis with Cox regression

	HR	95% CI	*P*-value
Age, years	1.027	(1.010–1.045)	0.002
Sex (male vs. female)	0.693	(0.443–1.083)	0.107
BMI, kg/m^2^	0.988	(0.941–1.037)	0.613
DBP, mmHg	0.995	(0.980–1.011)	0.555
HTN (yes vs. no)	0.844	(0.559–1.275)	0.421
DM (yes vs. no)	1.900	(1.341–2.692)	< 0.001
ACEI/ARB (yes vs. no)	0.958	(0.647–1.416)	0.828
B-blocker (yes vs. no)	1.410	(0.986–2.017)	0.060
CCB (yes vs. no)	1.140	(0.786–1.654)	0.490
Diuretics (yes vs. no)	0.880	(0.574–1.349)	0.558
eGFR*, mL/min /1.73m^2^	1.006	(1.002–1.010)	0.003
*Inflammatory score*			< 0.001
Score 1	1.615	(0.778–3.352)	0.198
Score 2	2.120	(1.029–4.366)	0.042
Score 3	4.649	(2.188–9.874)	< 0.001

* eGFR is calculated by Modification of Diet in Renal Disease (MDRD) equation. ACEI, angiotensin converting enzyme inhibitor; ARB, angiotensin receptor blocker; BMI, body mass index; CCB, calcium channel blocker; CI, confidence interval; DBP, diastolic blood pressure; DM, diabetes mellitus; eGFR, estimated glomerular filtration rate; HR, hazard ratio; HTN, hypertension

We also attempted to validate the findings by substituting MDRD equation with CKD-EPI equation to ascess renal function. A total of 134 patients with declined eGFR and 716 patients with maintaied eGFR were compared based on data derived from CKD-EPI equation. The results remained similar (Supplementary Tables 2 to 4).

## Discussion

In this study, we investigated the relationship between inflammatory markers and renal function decline in patients with chronic coronary syndrome. The major findings are (1) elevated hs-CRP, TNF-α and adiponectin were respectively associated with over 25% reduction in eGFR; (2) an inflammatory score depicting the collective effect of these biomarkers predicts the renal event. Our study provided additional support to the role of low-grade systemic inflammation as a major mechanism linking cardiovascular disease with impairment of renal function.

The role of inflammatory burden has been underscored in accompanying cardiovascular illness. Targeted anti-inflammatory therapies was proposed to improve clinical outcomes after ischemic events as well. Prospect to CANTOS (Canakinumab Anti-inflammatory Thrombosis Outcome Study), atherosclerosis has been recognized as an inflammatory disease and ameliorating the inflammatory status has been effective in reducing subsequent MACEs regardless of the lipid profile [12]. Evaluation based on STABILITY (Stabilization of Atherosclerotic Plaque by Initiation of Darapladib Therapy) trial database revealed inflammatory marker were positively correlated with the incidence of MACEs, and such phenomenon was unrelated to the renal function at baseline [13]. In addition, immunity adaptation with initiation of innate inflammatory process after AMI was demonstrated cytoprotective [14]. However, endpoints other than cardiovascular endpoints was not assessed in these trials. This study further reported the renal impact secondary to inflammatiory process in chronic coronary syndrome.

Inflammation has been established to compromise renal fucntion in various clinical background. A large-scale multicenter prognostic study with 5,061 subjects from ten nations reported that CRP levels were positively correlated with mortality rates in patients undergoing renal replacement therapy [15]. In the pre-dialysis population, the Chronic Renal Insufficiency Cohort study including 3,875 patients with stages II–IV CKD revealed that the elevation of inflammatory parameters, such as CRP, IL-6, and fibroblast growth factor 23, were independent predictive factors for mortality [16]. Based on the same cohort, Amdur et al. reported that the TNF-α level was also a remarkable prognostic factor [17]. Another prospective trial demonstrated that TNF-α predicted clinical outcomes and the development of diabetic nephropathy [18]. In conjunction with cyclooxygenase-2 and inducible nitric oxide synthase, TNF-α initiates the nuclear factor kappa-light-chain-enhancer of activated B cells (NF-κB) pathway. Deteriorated renal function also predisposes to the production of uremic toxins, thereby accentuating the levels of CRP and TNF-α through dysfunctional adipocytes and lymphocytes as well as upregulating the corresponding messenger ribonucleic acid expression. Besides, the level of adiponectin which contributes to and antagonizes inflammation in a context-dependent manner has been reported to be elevated in patients with CKD [19]. Interestingly, we proposed CRP, TNF-α, and adiponectin were of renal prognositic implication as well. In addition, the inflammation-based score system has been previously established to stratify in-hospital mortality risk in acute coronary event, but not in chronic coronary syndrome [20]. Inflammatory score was further conceptualized in this study to exemplify the aggregative effect of these biomarkers and better predict the renal effect in such population.

To evaluate renal impact, our study designated the cut-off at 25% of eGFR decline as a kidney event. This definition was endorsed by previous guideline and cohort investigation to represent subtle alteration in renal function [9, 21]. Our previous study also adapted such threshold to delineate the correlation between BP level and hypertensive nephropathy [22]. However, other more stringen changes of eGFR have been documented in the literature as well. In a prospective study by Puthamana et al. to delineate how inflammatory biomarkers impacts repair of kidney disease progression, a kidney event in participants without preexisting CKD at index hospitalization (eGFR ≥ 60 mL/min/1.73 m^2^), was defined as the combination of ≥ 25% reduction in eGFR and achieving CKD stage 3 or worse. For patients who have a lower eGFR at baseline (eGFR < 60 mL/min), a threshold of ≥ 50% reduction in eGFR was used instead [23]. Since the population in this study mostly exhibited intact renal function at baseline, the cut-off at 25% was therefore opted. Incorporation of other outcomes such as major adverse kidney event [24] consisting of CKD progression, initiation of long-term dialysis, and all-cause mortality will further recognize the renal effects of inflammatory burden.

Future perspectives of this study will attempt to attenuat the inflammatory status in patients with cardiovascular illness [25]. In the CANTOS, inhibition of interleukin-1β was associated with a significant decrease in hs-CRP and IL-6 levels as well as a reduction in vascular events. Subsequently, the LoDoCo2 (low-dose colchicine 2) trial [26] and COLCOT (Colchicine Cardiovascular Outcomes) trial [27] used colchicine as an anti-inflammatory agent and demonstrated effective secondary prevention of MACEs. A follow-up COLCHICINE-PCI randomized trial involving 400 subjects proposed that loading 1.8 mg oral colchicine before PCI significantly reduced the serum levels of hs-CRP and IL-6 [28]. The biomarker change, nevertheless, failed to ameliorate procedure-related myocardial injury or MACEs. However, the benefit was only observed in patients with preserved kidney function, probably because of its pharmacodynamics with dependence on renal excretion. These findings offer new insights into the impact of immunomodulatory therapies on patient outcomes, especially renal prognosis. Follow-up of this cohort study will propose the potential efficacy on renal function improvement by modulating inflammation.

This study has limitations. First, this non-randomized study is purely observational rather than interventional as only association but not causal relationship between inflammatory load and renal dysfunction could be concluded. Second, creatinine clearance was the only parameter used to assess renal function. Knowledge of other indicators such as urinary excretion of albumin and the application of different measures to assess eGFR, e.g. Cockcroft-Gault equation [29], will have enriched the assessment of the results. Third, the dynamic alterations in biomarkers were not documented, thereby compromising the interpretation of the longitudinal effect of the inflammatory burden in patients with chronic coronary syndrome. An extended follow-up period is also necessary to observe the eventual prognosis of renal function impairment. Forth, the single ethnicity of this study hampered the generation toward individuals with other racial backgrounds. Considering previous studies addressed the racial disparity in genetics as well as social determinants in the regulation of inflammation [30], trials including patients with more comprehensive genetic backgrounds are warranted. Fifth, the cohorts were enrolled from tertiary referral centers in Taiwan, which may not necessarily represent the characteristics of all patients with chronic coronary syndrome. Further studies are still indicated to extend the study to different hospital settings. Sixth, individuals who received non-steroidal anti-inflammatory drugs, steroids, or immunosuppressants were excluded. Although these patients may have a high inflammatory burden at baseline, the concomitant use of these medications might interfere with baseline inflammation burden and the outcomes of renal function decline. Seventh, the cut-off values were arbitrarily designated as 50th percentile to determine the presence of inflammatory burden. This cut-off may not be clinically meaningful or universally applicable. It would be valuable to determine the optimal thresholds for defining inflammatory burden in the future. Eighth, limited sample size of the cohort with eGFR decline hinders the elucidation of more subtle associations. However, to our knowledge this is by far the largest prospective study elucidating inflammation and renal function decline in chronic coronary syndrome. Finally, contrast-induced nephropathy might potentially confound the interrogation toward renal dysfunction after PCI. Since the study enrolled only clinically stable patients under medical treatment for at least one month and excluded those who anticipated to receive coronary or other cardiac interventions in the following one year, such effect is considered to be minimize [31].

In conclusion, our study demonstrated that a high inflammatory burden was correlated with renal function decline in patients with chronic coronary syndrome. Elevations in hs-CRP, TNF-α, and adiponectin levels were independent risk factors for significant eGFR decline. The aggregated effect, as presented by the inflammatory score, has prognostic implications for renal event. Future investigations will focus on appreciating the signalling pathways and thereby to identify potential therapeutic targets. A thorough understanding upon the role of inflammation will facilitate the advancement in holistic care for this population.

### Electronic supplementary material

Below is the link to the electronic supplementary material.

Supplementary Material 1

## Data Availability

The original datasets regarding this study is available from the corresponding author on reasonable request.

## List of abbreviations

AMI: Acute myocardial infarction
BMI: Body mass index
BP: Blood pressure
CAD: Coronary artery disease
CI: Confidence interval
CKD-EPI: Chronic Kidney Disease Epidemiology Collaboration
CKD: Chronic kidney disease
DM: Diabetes mellitus
eGFR: Estimated glomerular filtration rate
HR: Hazard ratio
hs-CRP: High sensitivity C-reactive protein
IL-6: Interleukin-6
Lp-PLA2: Lipoprotein-associated phospholipase A2
LVEF: Left ventricular ejection fraction
MACE: Major adverse cardiac event
MDRD: Modification of Diet in Renal Disease
MMP-9: Matrix metalloproteinase-9
NF-κB: Nuclear factor kappa-light-chain-enhancer of activated B cells
NT-pro BNP: N-terminal pro-brain natriuretic peptide
PCI: Percutaneous coronary intervention
TNF-α: Tumor necrosis factor-α

## References

[1] Masoudi FA, Ponirakis A, de Lemos JA, Jollis JG, Kremers M, Messenger JC (2017). Trends in U.S. Cardiovascular Care: 2016 Report from 4 ACC National Cardiovascular Data Registries. J Am Coll Cardiol.

[2] Stark K, Massberg S (2021). Interplay between inflammation and Thrombosis in cardiovascular pathology. Nat Rev Cardiol.

[3] Stuveling EM, Hillege HL, Bakker SJ, Gans RO, De Jong PE, De Zeeuw D (2003). C-reactive protein is associated with renal function abnormalities in a non-diabetic population. Kidney Int.

[4] Sauriasari R, Sakano N, Wang DH, Takaki J, Takemoto K, Wang B (2010). C-reactive protein is associated with cigarette smoking-induced hyperfiltration and proteinuria in an apparently healthy population. Hypertens Res.

[5] Gao J, Wang A, Li X, Li J, Zhao H, Zhang J (2020). The cumulative exposure to high-sensitivity C-reactive protein predicts the risk of chronic kidney Diseases. Kidney Blood Press Res.

[6] Leu HB, Yin WH, Tseng WK, Wu YW, Lin TH, Yeh HI (2017). Identification of new biosignatures for clinical outcomes in stable coronary artery Disease - the study protocol and initial observations of a prospective follow-up study in Taiwan. BMC Cardiovasc Disord.

[7] Schwandt A, Denkinger M, Fasching P, Pfeifer M, Wagner C, Weiland J (2017). Comparison of MDRD, CKD-EPI, and Cockcroft-Gault equation in relation to measured glomerular filtration rate among a large cohort with Diabetes. J Diabetes Complications.

[8] Inker LA, Eneanya ND, Coresh J, Tighiouart H, Wang D, Sang Y, Chronic Kidney Disease Epidemiology Collaboration (2021). New creatinine- and cystatin C-based equations to estimate GFR without race. N Engl J Med.

[9] Inker LA, Astor BC, Fox CH, Isakova T, Lash JP, Peralta CA (2014). KDOQI US commentary on the 2012 KDIGO clinical practice guideline for the evaluation and management of CKD. Am J Kidney Dis.

[10] Lemieux I, Pascot A, Prud’homme D, Alméras N, Bogaty P, Nadeau A (2001). Elevated C-reactive protein: another component of the atherothrombotic profile of abdominal obesity. Arterioscler Thromb Vasc Biol.

[11] Mathieu P, Lemieux I, Després JP (2010). Obesity, inflammation, and cardiovascular risk. Clin Pharmacol Ther.

[12] Ridker PM, Everett BM, Thuren T, MacFadyen JG, Chang WH, Ballantyne C (2017). CANTOS Trial Group. Antiinflammatory therapy with canakinumab for atherosclerotic Disease. N Engl J Med.

[13] Batra G, Ghukasyan Lakic T, Lindbäck J, Held C, White HD, Stewart RAH (2021). STABILITY investigators. Interleukin 6 and cardiovascular outcomes in patients with chronic Kidney Disease and chronic coronary syndrome. JAMA Cardiol.

[14] Santos-Zas I, Lemarié J, Tedgui A, Ait-Oufella H (2019). Adaptive immune responses contribute to post-ischemic cardiac remodeling. Front Cardiovasc Med.

[15] Bazeley J, Bieber B, Li Y, Morgenstern H, de Sequera P, Combe C (2011). C-reactive protein and prediction of 1-year mortality in prevalent hemodialysis patients. Clin J Am Soc Nephrol.

[16] Munoz Mendoza J, Isakova T, Cai X, Bayes LY, Faul C, Scialla JJ (2017). CRIC Study investigators. Inflammation and elevated levels of fibroblast growth factor 23 are Independent risk factors for death in chronic Kidney Disease. Kidney Int.

[17] Amdur RL, Feldman HI, Gupta J, Yang W, Kanetsky P, Shlipak M (2016). CRIC Study investigators. Inflammation and progression of CKD: the CRIC Study. Clin J Am Soc Nephrol.

[18] Neirynck N, Glorieux G, Schepers E, Verbeke F, Vanholder R (2015). Soluble Tumor necrosis factor receptor 1 and 2 predict outcomes in advanced chronic Kidney Disease: a prospective cohort study. PLoS ONE.

[19] Song SH, Oh TR, Choi HS, Kim CS, Ma SK, Oh KH (2020). High serum adiponectin as a biomarker of renal dysfunction: results from the KNOW-CKD study. Sci Rep.

[20] González-Pacheco H, Bojalil R, Amezcua-Guerra LM, Sandoval J, Eid-Lidt G, Arias-Mendoza A (2019). Derivation and validation of a simple inflammation-based risk score system for predicting in-hospital mortality in acute coronary syndrome patients. J Cardiol.

[21] Kovesdy CP, Coresh J, Ballew SH, Woodward M, Levin A, Naimark DM (2016). CKD Prognosis Consortium. Past decline versus current eGFR and subsequent ESRD risk. J Am Soc Nephrol.

[22] Kao TW, Huang CC, Chen JW (2020). Optimal blood pressure for the prevention of hypertensive Nephropathy in nondiabetic hypertensive patients in Taiwan. J Clin Hypertens.

[23] Puthumana J, Thiessen-Philbrook H, Xu L, Coca SG, Garg AX, Himmelfarb J (2021). Biomarkers of inflammation and repair in Kidney Disease progression. J Clin Invest.

[24] McKown AC, Wang L, Wanderer JP, Ehrenfeld J, Rice TW, Bernard GR (2017). Predicting major adverse kidney events among critically ill adults using the electronic health record. J Med Syst.

[25] Kao TW, Huang CC (2022). Inflammatory burden and immunomodulative therapeutics of Cardiovascular Diseases. Int J Mol Sci.

[26] Nidorf SM, Fiolet ATL, Mosterd A, Eikelboom JW, Schut A, Opstal TSJ (2020). LoDoCo2 trial investigators. Colchicine in patients with chronic coronary Disease. N Engl J Med.

[27] Tardif JC, Kouz S, Waters DD, Bertrand OF, Diaz R, Maggioni AP (2019). Efficacy and safety of low-dose colchicine after Myocardial Infarction. N Engl J Med.

[28] Shah B, Pillinger M, Zhong H, Cronstein B, Xia Y, Lorin JD (2020). Effects of acute colchicine administration prior to percutaneous coronary intervention: COLCHICINE-PCI randomized trial. Circ Cardiovasc Interv.

[29] Rivera-Caravaca JM, Ruiz-Nodar JM, Tello-Montoliu A, Esteve-Pastor MA, Quintana-Giner M, Véliz-Martínez A (2018). Disparities in the estimation of glomerular filtration rate according to Cockcroft-Gault, modification of Diet in Renal Disease-4, and Chronic Kidney Disease Epidemiology Collaboration Equations and relation with outcomes in patients with acute coronary syndrome. J Am Heart Assoc.

[30] Kocarnik JM, Pendergrass SA, Carty CL, Pankow JS, Schumacher FR, Cheng I (2014). Multiancestral analysis of inflammation-related genetic variants and C-reactive protein in the population architecture using genomics and epidemiology study. Circ Cardiovasc Genet.

[31] Rear R, Bell RM, Hausenloy DJ (2016). Contrast-induced Nephropathy following angiography and cardiac interventions. Heart.

