# Supplementary material for: Inflammation and renal function decline in chronic coronary syndrome: a prospective multicenter cohort study
Source: BMC Cardiovasc Disord. 2023 Nov 16;23:564. doi: 10.1186/s12872-023-03565-5 (PMC10655285; doi:10.1186/s12872-023-03565-5)
Supplement: Supplementary file 1 — Supplementary Material 1 [file 12872_2023_3565_MOESM1_ESM.docx]

**Supplementary files**

**Inflammation and Renal Function Decline in Chronic Coronary Syndrome: A Prospective Multicenter Cohort Study**

Ting-Wei Kao^1^, Chin-Chou Huang^2,3,4,*^, Hsin-Bang Leu^4,5^, Wei-Hsian Yin^4,6^, Wei-Kung Tseng^7,8^, Yen-Wen Wu^4,9^, Tsung-Hsien Lin^10^, Hung-I Yeh^11^, Kuan-Cheng Chang^12,13^, Ji-Hung Wang^14^, Chau-Chung Wu^15,16^, Jaw-Wen Chen^3,5^

Table of contents

**Supplementary Table 1.** Angiographic characteristics of enrolled subjects

**Supplementary Table 2.** Baseline characteristics of enrolled subjects (CKD-EPI equation)

**Supplementary Table 3**. Association between levels of inflammatory markers and renal events by multivariate analysis with Cox regression (CKD-EPI equation)

**Supplementary Table 4.** Inflammatory scores and renal function decline by multivariate analysis with Cox regression (CKD-EPI equation)

**Supplementary Table 1** Angiographic characteristics of enrolled subjects

|  | **All**  **(n=687)** | **eGFR* decline**  **(n=110)** | **eGFR* maintained**  **(n=577)** | ***P-value*** |
| --- | --- | --- | --- | --- |
| LM, n(%) | 57 (8.3%) | 9 (8.2%) | 48 (8.3%) | 0.962 |
| LAD, n(%) | 546 (79.5%) | 95 (86.4%) | 451 (78.2%) | 0.051 |
| LCX, n(%) | 360 (52.4%) | 60 (54.5%) | 300 (52.0%) | 0.623 |
| RCA, n(%) | 416 (60.6%) | 66 (60.0%) | 350 (60.7%) | 0.897 |

* eGFR is calculated by Modification of Diet in Renal Disease (MDRD) equation. LAD, left anterior descending artery; LCX, left circumflex artery; LM, left main artery; RCA, right coronary artery.

**Supplementary Table 2.** Baseline characteristics of enrolled subjects (CKD-EPI equation)

|  | **All**  **(n=850)** | **eGFR* decline**  **(n=134)** | **eGFR* maintained**  **(n=716)** | ***P-value*** |
| --- | --- | --- | --- | --- |
| Age, years | 66.3 ± 12.4 | 70.4 ± 12.8 | 65.5 ± 12.1 | <0.001 |
| Male, n(%) | 729 (85.8%) | 109 (81.3%) | 620 (86.6%) | 0.110 |
| BMI, kg/m2 | 26.2 ± 3.6 | 25.8 ± 3.6 | 26.3 ± 3.6 | 0.185 |
| SBP, mmHg | 128.7 ± 16.4 | 130.1 ± 17.9 | 128.5 ± 16.1 | 0.350 |
| DBP, mmHg | 73.9 ± 12.2 | 71.0 ± 13.9 | 74.4 ± 11.8 | 0.008 |
| HTN, n(%) | 560 (65.9%) | 93 (69.4%) | 467 (65.2%) | 0.349 |
| DM, n(%) | 306 (36.0%) | 65 (48.5%) | 241 (33.7%) | 0.001 |
| Smoking, n(%) | 477 (56.1%) | 71 (53.0%) | 406 (56.7%) | 0.426 |
| ACEI/ARB, n(%) | 555 (65.3%) | 93 (69.4%) | 462 (64.5%) | 0.276 |
| B-blocker, n(%) | 508 (59.8%) | 84 (62.7%) | 424 (59.2%) | 0.452 |
| CCB, n(%) | 343 (40.4%) | 62 (46.3%) | 281 (39.2%) | 0.128 |
| Diuretics, n(%) | 152 (17.9%) | 33 (24.6%) | 119 (16.6%) | 0.026 |
| Statins, n(%) | 631 (74.2%) | 97 (72.4%) | 534 (74.6%) | 0.594 |
| LVEF < 40%, n(%) | 49 (5.8%) | 10 (7.5%) | 39 (5.4%) | 0.358 |
| Creatinine, mg/dL | 1.1 ± 0.3 | 1.2 ± 0.4 | 1.1 ± 0.3 | <0.001 |
| eGFR*, mL/min /1.73m^2^ | 73.0 ± 20.8 | 66.0 ± 23.9 | 74.3 ± 19.9 | <0.001 |
| Uric acid, mg/dL | 6.5 ± 1.6 | 6.8 ± 1.8 | 6.4 ± 1.6 | 0.012 |
| hs-CRP, mg/dL | 0.3 ± 0.9 | 0.5 ± 1.7 | 0.3 ± 0.7 | 0.025 |
| TNF-α, pg/mL | 4.0 ± 4.7 | 4.4 ± 5.4 | 3.9 ± 4.5 | 0.289 |
| Adiponectin, ng/mL | 8096.8 ± 13341.3 | 11150.0 ± 18053.9 | 7525.4 ± 12192.0 | 0.004 |
| NT-pro BNP, pg/mL | 418.0 ± 955.3 | 756.7 ± 1931.6 | 354.6 ± 603.4 | <0.001 |
| Follow up duration, years | 3.5 ± 1.9 | 3.7 ± 1.8 | 3.5 ± 1.9 | 0.281 |

* eGFR is calculated by CKD-EPI (Chronic Kidney Disease Epidemiology Collaboration) equation. ACEI, angiotensin converting enzyme inhibitor; ARB, angiotensin receptor blocker; BMI, body mass index; CCB, calcium channel blocker; DBP, diastolic blood pressure; DM, diabetes mellitus; eGFR, estimated glomerular filtration rate; HTN, hypertension; hs-CRP, high-sensitivity C-reactive protein; LVEF, left ventricular ejection fraction; NT-pro BNP, N-terminal pro-brain natriuretic peptide; SBP, systolic blood pressure; TNF-α, tumor necrosis factor-α.

**Supplementary Table 3**. Association between levels of inflammatory markers and renal events by multivariate analysis with Cox regression (CKD-EPI equation)

|  | **HR (95% CI)** | ***P-value*** |  | **HR (95% CI)** | ***P-value*** |  | **HR (95% CI)** | ***P-value*** |  | **HR (95% CI)** | ***P-value*** |
| --- | --- | --- | --- | --- | --- | --- | --- | --- | --- | --- | --- |
| Age, years | 1.024 (1.004–1.044) | 0.019 | Age, years | 1.021 (1.001–1.041) | 0.036 | Age, years | 1.022 (1.002–1.042) | 0.029 | Age, years | 1.022 (1.003–1.043) | 0.027 |
| Sex  (male vs. female) | 0.755 (0.476–1.197) | 0.231 | Sex  (male vs. female) | 0.780 (0.493–1.235) | 0.289 | Sex  (male vs. female) | 0.781 (0.494–1.234) | 0.290 | Sex  (male vs. female) | 0.760 (0.479–1.206) | 0.244 |
| BMI, kg/m^2^ | 0.986 (0.936–1.038) | 0.586 | BMI, kg/m^2^ | 0.985 (0.937–1.037) | 0.572 | BMI, kg/m^2^ | 0.985 (0.936–1.037) | 0.569 | BMI, kg/m^2^ | 0.987 (0.938–1.039) | 0.621 |
| DBP, mmHg | 0.986 (0.970–1.002) | 0.087 | DBP, mmHg | 0.988 (0.972–1.004) | 0.126 | DBP, mmHg | 0.988 (0.972–1.004) | 0.131 | DBP, mmHg | 0.989 (0.974–1.005) | 0.196 |
| HTN  (yes vs. no) | 0.905 (0.588–1.393) | 0.650 | HTN  (yes vs. no) | 0.966 (0.626–1.491) | 0.876 | HTN  (yes vs. no) | 0.928 (0.603–1.428) | 0.735 | HTN  (yes vs. no) | 0.905 (0.588–1.393) | 0.650 |
| DM  (yes vs. no) | 1.946 (1.348–2.808) | <0.001 | DM  (yes vs. no) | 1.889 (1.312–2.719) | 0.001 | DM  (yes vs. no) | 1.883 (1.308–2.710) | 0.001 | DM  (yes vs. no) | 1.912 (1.326–2.757) | 0.001 |
| ACEI/ARB  (yes vs. no) | 0.854 (0.568–1.282) | 0.445 | ACEI/ARB  (yes vs. no) | 0.831 (0.554–1.245) | 0.369 | ACEI/ARB  (yes vs. no) | 0.829 (0.552–1.245) | 0.366 | ACEI/ARB  (yes vs. no) | 0.863 (0.573–1.302) | 0.483 |
| B-blocker  (yes vs. no) | 1.175 (0.810–1.706) | 0.396 | B-blocker  (yes vs. no) | 1.179 (0.813–1.709) | 0.385 | B-blocker  (yes vs. no) | 1.233 (0.850–1.789) | 0.271 | B-blocker  (yes vs. no) | 1.217 (0.836–1.770) | 0.305 |
| CCB  (yes vs. no) | 1.147 (0.782–1.684) | 0.483 | CCB  (yes vs. no) | 1.175 (0.802–1.722) | 0.407 | CCB  (yes vs. no) | 1.168 (0.796–1.713) | 0.428 | CCB  (yes vs. no) | 1.229 (0.836–1.806) | 0.294 |
| Diuretics  (yes vs. no) | 0.899 (0.572–1.415) | 0.646 | Diuretics  (yes vs. no) | 0.976 (0.627–1.518) | 0.913 | Diuretics  (yes vs. no) | 0.961 (0.618–1.494) | 0.859 | Diuretics  (yes vs. no) | 0.975 (0.626–1.519) | 0.912 |
| eGFR*, mL/min /1.73m^2^ | 0.992 (0.982–1.002) | 0.127 | eGFR*, mL/min /1.73m^2^ | 0.992 (0.982–1.002) | 0.128 | eGFR*, mL/min /1.73m^2^ | 0.992 (0.982–1.002) | 0.105 | eGFR*, mL/min /1.73m^2^ | 0.993 (0.983–1.003) | 0.196 |
| hs-CRP | 1.209 (1.095–1.336) | <0.001 | TNF-α | 1.015 (0.985–1.047) | 0.318 | Adiponectin x10^3^ | 1.009 (1.001–1.017) | 0.021 | NT-pro BNP x 10^3^ | 1.077 (1.006–1.152) | 0.032 |

* eGFR is calculated by CKD-EPI (Chronic Kidney Disease Epidemiology Collaboration) equation. ACEI, angiotensin converting enzyme inhibitor; ARB, angiotensin receptor blocker; BMI, body mass index; CCB, calcium channel blocker; CI, confidence interval; DBP, diastolic blood pressure; DM, diabetes mellitus; eGFR, estimated glomerular filtration rate; HTN, hypertension; HR, hazard ratio; hs-CRP, high-sensitivity C-reactive protein; NT-pro BNP, N-terminal pro-brain natriuretic peptide; TNF-α, tumor necrosis factor-α.

**Supplementary Table 4.** Inflammatory scores and renal function decline by multivariate analysis with Cox regression (CKD-EPI equation)

|  | **HR** | **95% CI** | ***P*-value** |
| --- | --- | --- | --- |
| Age, years | 1.020 | (1.000–1.040) | 0.052 |
| Sex (male vs. female) | 0.732 | (0.462–1.161) | 0.185 |
| BMI, kg/m^2^ | 0.984 | (0.935–1.035) | 0.520 |
| DBP, mmHg | 0.990 | (0.974–1.005) | 0.196 |
| HTN (yes vs. no) | 0.936 | (0.608–1.442) | 0.765 |
| DM (yes vs. no) | 1.975 | (1.371–2.843) | <0.001 |
| ACEI/ARB (yes vs. no) | 0.852 | (0.569–1.274) | 0.435 |
| B-blocker (yes vs. no) | 1.242 | (0.859–1.796) | 0.250 |
| CCB (yes vs. no) | 1.074 | (0.731–1.579) | 0.717 |
| Diuretics (yes vs. no) | 0.956 | (0.616–1.483) | 0.840 |
| eGFR*, mL/min /1.73m^2^ | 0.995 | (0.985–1.005) | 0.307 |
| Inflammatory score | | | <0.001 |
| Score 1 | 1.416 | (0.677–2.959) | 0.356 |
| Score 2 | 1.789 | (0.861–3.717) | 0.119 |
| Score 3 | 3.644 | (1.692–7.847) | 0.001 |

*eGFR is calculated by CKD-EPI (Chronic Kidney Disease Epidemiology Collaboration) equation. ACEI, angiotensin converting enzyme inhibitor; ARB, angiotensin receptor blocker; BMI, body mass index; CCB, calcium channel blocker; CI, confidence interval; DBP, diastolic blood pressure; DM, diabetes mellitus; eGFR, estimated glomerular filtration rate; HR, hazard ratio; HTN, hypertension.
